# Vitamin D and omega-3 fatty acids attenuate MSG-induced neurodegeneration by modulating tau pathology, neuroinflammation, and VDR expression in rats

**DOI:** 10.1038/s41598-025-06109-3

**Published:** 2025-06-20

**Authors:** Rasha Z. Alshahawy, Sally M. Safwat, Sanad S. El-Kholy, Soheir F. El-basiony, Sara El-Desouky, Soheir M. Helmi

**Affiliations:** 1 Department of Medical Physiology, Faculty of Medicine, Kafr-Elsheikh University, Kafr-Elsheikh, Egypt; 2 Department of Medical Physiology, Faculty of Medicine, Mansoura University, Mansoura, Egypt; 3 Medical Experimental Research Center (MERC), Faculty of Medicine, Mansoura University, Mansoura, Egypt

**Keywords:** MSG, Vitamin D, Omega-3 PUFAs, VDRs, Neuroinflammation, Tau pathology, Neuroscience, Physiology

## Abstract

Monosodium glutamate (MSG)-induced excitotoxicity is a major factor contributing to cognitive decline and neurodegeneration. Given the well-established roles of vitamin D (Vit D) and omega-3 polyunsaturated fatty acids (N-3 PUFAs), especially eicosapentaenoic acid (EPA) and docosahexaenoic acid (DHA), in neuroprotection, the present study aimed at analyzing their possible neuroprotective efficacy against MSG-induced neurotoxicity in rats, concerning the behavioral performance, hippocampal histological integrity, and pathological protein accumulation, along with determination of the inflammatory marker levels and mRNA expression of vitamin D receptors (VDR) and other neurodegeneration-related genes. Fifty male Sprague Dawley rats were randomly allocated to a control, an MSG, and three treatment groups that received MSG and either Vit D or N-3 PUFA supplements in combinations or alone for 4 weeks. At the end of the study, five behavioral tests were conducted to assess cognitive functions, motor activity, and anxiety-related behaviors, and hippocampal tissues were analyzed for tau pathology, neuroinflammation, expression of VDR, and neurodegeneration-related markers. The results demonstrated that supplementation with Vit D (1 mcg/kg) and N-3 PUFAs (300 mg/kg EPA + DHA) profoundly attenuated MSG-induced neurodegeneration. The combined therapy decreased neuronal damage caused by MSG by 87% and tau pathology by 83%. The combined treatment further suppressed pro-inflammatory cytokines (TNF-α: 52%; IL-6: 65%) and elevated anti-inflammatory IL-10 by 2.8-fold, demonstrating a dual anti-inflammatory action. A major upregulation of hippocampal VDR by 4.6-fold was noted, with stabilization of calcium homeostasis and normalization of caspase-3 and α-synuclein expression. Our findings confirm that Vit D and N-3 PUFAs exhibit substantial synergistic neuroprotective abilities that might be mediated through synergistic VDR upregulation, providing a promising dietary intervention against MSG-induced excitotoxicity and highlighting their broader implications for supporting cognitive health and mitigating the adverse effects of other neurotoxins.

## Introduction

Accumulating evidence indicates that exposure to neurotoxic chemicals, especially in early life, can lead to a variety of neurodevelopmental and neurological diseases, as growing brains are more sensitive to insults than adult brains^1,2^. The hippocampus, in particular, is a complex region in the limbic system known to play a crucial role in the processing of cognitive functions, particularly learning and memory, as well as mood regulation and affective behaviors. Additionally, the hippocampus has been shown to be more susceptible to neurotoxic damage, even more severely and earlier than other brain structures^3^. Monosodium glutamate (MSG) is the sodium salt of glutamic acid and is one of the most commonly used food additives in processed foods^4^. Although the Food and Drug Administration (FDA) claimed that MSG is a harmless substance, several animal studies have demonstrated the negative consequences of excessive MSG use^5^. Following intake, MSG dissociates in the body into glutamate and sodium ions^4^. Glutamate is the main excitatory amino acid in the mammalian brain^6^, where glutamate receptors, such as N-methyl-d-aspartate (NMDA) receptors, are widely distributed, particularly in the hypothalamus, hippocampus, and amygdala, as they play fundamental roles in neuronal signaling, affecting several key brain activities, such as synaptic stabilization, cognition, memory, learning, and cellular metabolism^7^. Importantly, extracellular glutamate levels are tightly regulated, as their overabundance at synapses are inked to glutamate-induced excitotoxicity, a condition caused by elevated extracellular brain glutamate concentrations combined with excessive glutamatergic stimulation, resulting in surges in intracellular calcium levels and the initiation of neurotoxic signaling pathways, that are implicated in several neurological pathologies, including neurodegenerative disorders^6,8^. Experimentally, excessive administration of MSG leads to elevated amounts of glutamic acid in the blood, resulting in glutamate overload that bypasses blood-brain barrier regulation, overactivating NMDA receptors, and triggering cascades that disrupt neuronal homeostasis^5,9^.

In the same context, MSG consumption has been reported to cause neurodegeneration and cognitive deficits in rats by increasing cation levels, oxidative stress, inflammatory cytokine levels, acetylcholinesterase, and LDH activity, and decreasing GABA levels via mechanisms involving its excitotoxic pathways^10^. Additionally, the literature revealed that MSG administration has been involved in the earlier development of AD-like pathology, including increased amyloid beta (Aβ) and phosphorylated tau protein (p-tau) levels^11^. Furthermore, several previous studies have shown that aberrant aggregation of α-synuclein, the principal structural component of the Lewy body in Parkinson’s disease (PD), is closely associated with glutamic acid excitatory toxicity, which in turn could be mediated by excess MSG exposure^12–14^. Similarly, neonatal MSG exposure is connected to hyperglycemia, AD-like learning, and memory difficulties; decreased dendritic spine density; elevated p-tau; and abnormal hippocampal synaptic protein synthesis^15^, which might constitute a potential neuropathological pathway of excessive dietary MSG consumption.

In addition, the toxic effects of MSG have expanded to different organs and have been demonstrated to be involved in hepatotoxicity^16^, renal toxicity^17^, obesity^18^, diabetes^19^, and reproductive dysfunction^20^, raising questions about the risks of MSG ingestion in humans and prompting the exploration of dietary interventions that could mitigate these adverse effects of MSG consumption.

In contrast to MSG, vitamin D (Vit D) and omega-3 polyunsaturated fatty acids (N-3 PUFAs) have long been recognized as supporting agents for brain health^21,22^. Vit D works through numerous neuroprotective mechanisms, affects brain development and maturation, and supports antioxidant and anti-inflammatory pathways^23^. Vit D roles in maintaining central nervous system homeostasis relies on its interaction with the vitamin D receptor (VDR), a nuclear receptor widely expressed in the brain regions, such as in the hippocampus, cortex, and substantia nigra^24^. VDR activation by Vit D is characterized by genomic signaling and is consequently responsible for regulating the transcription of genes necessary for neuronal survival, such as those involved in calcium homeostasis (e.g., calbindin-D28k) and antioxidant defense (e.g., glutathione and superoxide dismutase(SOD) )^25,26^. Besides, Vit D blocks neuroinflammation through inhibition of Nuclear factor-κB (NF-κB) signaling, decelerating pro-inflammatory cytokines while increasing the production of anti-inflammatory cytokines such as IL-10, as proven in models of neurodegeneration^27^. Non-genomic pathways enhance its protective role: Vit D has shown to stabilize mitochondrial function, relieve oxidative stress by activating the Nrf2 pathway, and modulate synaptic plasticity through stimulation of neurotrophic factors such as brain-derived neurotrophic factor (BDNF) and glial cell line-derived neurotrophic factor (GDNF)^28^. All these mechanisms help in the mitigation of excitotoxicity, protein aggregation, and apoptotic cascades, regarding Vit D as an important player in the regulation of brain resilience against metabolic and inflammatory insults^29^.

Similarly, many studies have shown that increased consumption of N-3 PUFAs supports brain health, improves cognitive well-being, and learning and memory abilities^30^. The neuroprotective effects of N-3PUFAs, particularly docosahexaenoic acid (DHA) and eicosapentaenoic acid (EPA), are believed to function via their incorporation into neuronal membranes and modulation of key signaling pathways^31^. Structurally, DHA exists as about 30% of the phospholipids within synaptic membranes, where it enhances membrane fluidity and thereby facilitates neurotransmission, receptor clustering, and synaptic vesicle fusion^32^. From their structural support, DHA leads the way to synaptic plasticity via increasing dendritic arborization, stimulation of neurogenesis, and long-term potentiation (LTP) enhancement through modulation of NMDA receptor activity and increasing BDNF synthesis^30,33^. In addition to their structural functions, N-3 PUFA exert anti-inflammatory properties through activation of Peroxisome proliferator-activated receptor gamma (PPAR-γ), contributing to the downregulation of NF-κB-induced cytokine production (e.g., TNF-α, IL-6)^34–36^, and the upregulation of microglial phagocytosis of Aβ aggregates^37^. At the same time, N-3 PUFAs enhance the Nrf2 antioxidant pathway, resulting in relief of oxidative stress via transcriptionally upregulating glutathione and scavenging the reactive oxygen species^38^. Additionally, EPA and DHA are precursors to specialized pro-resolving mediators such as resolvins and protectins, which mediate the resolution of chronic neuroinflammation and tissue repair^37^. Furthermore, N-3 PUFAs counteract tau hyperphosphorylation, resulting in reduced neurofibrillary tangle formation^39^. In a nutshell, these numerous concurrent N-3 PUFAs-mediated mechanisms increase neuronal robustness against neurodegenerative cascades and can be regarded as key dietary modulators of brain health^30^.

Together, there is increasing scientific data on the potential benefit of Vit D and N-3 PUFA supplementation in neurodegenerative illnesses such as PD and AD^23,30^; however, the combined effectiveness of these treatments against MSG-induced neurotoxicity remains underinvestigated. Therefore, this study aimed to investigate the combined neuroprotective effects of Vit D and N-3 PUFAs, to elucidate how their complementary mechanisms could mitigate glutamate-induced excitotoxicity, advancing dietary strategies for neuroprotection.

## Materials and methods

The experimental protocol was approved by the Mansoura University Animal Care and Use Committee (MU-ACUC), approval code: MU-ACUC (MED.PhD.23.06.2). All applicable international, national, and institutional ethical guidelines for the care and use of animals were followed. All procedures were performed under ARRIVE guidelines and followed the U.K. Animals ACT, of 1986.

### Experimental animals

This study was conducted with 50 male Sprague–Dawley rats aged 4–5 weeks and weighing 40–60 g. Rats were purchased from the animal house of the Medical Experimental Research Center (MERC), Faculty of Medicine, Mansoura University, and they were housed there in cleaned and good aerated metal cages (5 per cage). They were allowed free access to rodent laboratory food and water throughout the experiment. The housing facility was kept under constant laboratory conditions (24 ± 2 °C), with a 12-hour light, 12-hour dark cycle under a controlled humidity environment. All practical work and laboratory procedures have considered the precautions of biosecurity and biosafety for research work in laboratories^40^.

### Experimental design

The rats were randomly allocated to one of five groups (*n* = 10 each).

I. The control group received a single daily oral dose of saline as a negative control for 4 weeks.
II. MSG- group (MSG): Rats received a series of oral doses of MSG (purchased from El-Goumhouria Co. Cairo, Egypt) and 2 g/kg body weight 10% aqueous solution once every other day for 10 days^41^.
III. Vitamin D-treated group (MSG + Vit D): Rats received an oral dose of MSG once every other day for 10 days, accompanied by supplementation with Vit D (calcitriol; purchased from Medical Union Pharmaceuticals (MUP) Co. Cairo, Egypt) at 1 mcg/kg BW once/day for 4 weeks^42,43^.
IV. Omega-3 PUFA-treated group (MSG + N-3): Rats received an oral dose of MSG once every other day for 10 days accompanied by supplementation with long-chain N-3 PUFAs (eicosapentaenoic acid (EPA) and docosahexaenoic acid (DHA) purchased from NOW Foods Egypt Co. Cairo, Egypt, 300 mg/kg BW (once/day) for 4 weeks^44^.
V. In the combination therapy group (MSG + Vit D + N-3), the rats received an oral dose of MSG once every other day for 10 days, followed by supplementation with 300 mg/kg BW N-3 PUFAs (EPA and DHA)^44^ + Vit D (calcitriol (1 mcg/kg BW)) once/day for 4 weeks^42^.

### Behavioral tests

Behavioral tests were conducted to evaluate various areas of cognitive function, including emotionality (dark–light and open field tests), spatial working memory (Morris water maze (MWM) and T-maze), exploratory activity (novel object recognition (NOR)), and motor activity and coordination (open field test)^45^. All tests were carried out at the Department of Medical Physiology, Mansura University, Egypt, from 9:00 a.m. to 2:00 p.m. in a quiet observation room with natural light. The rats were acclimated in the room for 30 min before the beginning of the tests, which were recorded by the videotape program Cyber Link You Cam 365 (version: 10.1.2717.0). The recorded activity was stored on a computer and subsequently analyzed with the software tracking system ANY maze™ (version 7.44). Some tests’ measurements have been documented manually and their related parameters were analyzed using the appropriate statistical program.

#### Open field test

Open field test is potentially sensitive to both motor activity and exploration in addition to its use as a measure of anxiety. After acclimatization to the environment, the rats were placed in an open field consisting of a 100 × 100 cm box with 21 cm high walls around it. The floor was divided into 4 × 4 areas. The rats were placed individually in the center, always facing the same direction, and were video recorded for 5 min. The following parameters of locomotor activity were measured: total distance traveled, average speed of movement, number of crossings, rearing, and grooming behavior, in addition to anxiety-related measurements (number of entries and time spent in the central zone)^46^.

#### Novel object recognition test (NOR)

NOR is a commonly used behavioral assay for the investigation of various aspects of exploration activities, learning, and memory in rodents. The test is composed of three phases: the habituation, training, and test phases. These tests were carried out on 3 consecutive days for 5 min each. The habituation phase was carried out in an empty open field arena (24 h before short-term memory was evaluated to reduce anxiety and stress). The training and test phases were carried out 24 h apart to assess short- and long-term memory. In the training phase, the rats were allowed to recognize two identical cuboid objects located 15 cm from the wall and 60 cm apart. In the test phase, a novel cone-shaped object replaced one of the familiar objects. The time spent per second by each rat to explore the objects was recorded. The recognition or preference index was then calculated as the time spent exploring the novel object divided by the total exploration time*100^46^.

#### Spontaneous alternation T-maze test

The T-maze test is used to assess short-term spatial working memory. As rodents have a strong exploration drive, it can be utilized as motivation to perform a short-term spatial working memory test in an enclosed T-maze^46^. The spontaneous alternation T-maze is based on a rat’s inherent preference for investigating a novel arm over a familiar one, motivating them to alternate their choice of the target arm^47^. The test consists of one sample trial (T0) and several test trials carried out in a single session. At T0, the rats were released into the stem of the T-maze (the start arm), facing away from the decision point (arm junction), and permitted to freely pick one of the two lateral goal arms to explore. A sliding door is lowered after the rat entrance, and the animal is confined to the chosen arm for 10 s before being removed and replaced in the start arm again, with all doors raised for the following test trial. A set of six test trials (T1-T6) is then performed, the choice latencies for entering the arms during the test session from T0-T6 are recorded, and the percentages of correct alternations across the test trials are calculated as an index of working memory via the following formula: (total number of correct alternations/6) * 100^48^.

#### Morris water maze test (MWM)

To examine hippocampus-dependent spatial learning and memory, the MWM test was conducted. Performance was evaluated in a 110-cm-diameter cylindrical water pool, as originally reported by Richard Morris, with minor modifications^49^. During the training trials, a platform was placed in a fixed location, four bright and colorful cards were positioned on the wall in each quadrant, and neither the pool nor its surroundings were altered. The lighting and water temperature were consistent throughout the test. The test was carried out on a single day and consisted of three pretraining trials, twelve training trials, and one prob trial for each rat.

For pretraining, the platform was exposed one inch above the surface of the clean water. Each animal conducted three consecutive trials, starting in a different direction, once from each quadrant apart from the one where the platform was placed (the target quadrant). The rats were placed gently, facing the wall of the arena, allowed to swim to the visible platform for up to 60s, and allowed to sit there for 15s.

For training trials, the pool was overfilled, so the platform was submerged one inch beneath the water, which has been modified to be opaque using starch. The rats had to swim to a hidden platform and rely on clues from outside the maze. Each animal had 12 trials, four for each starting direction apart from the target one; all the rats completed the same trial number before moving on to the following trial. The time taken by each rat to reach the platform was recorded and reported for each trial as the latency period. The rats that failed to reach the platform within 60 s were gently directed (without being removed from the water) to the platform and left there for 15 s (the latency period for those mice was recorded as 60 s).

For the probe trial, after all the animals had completed 12 trials, they performed one probe trial, in which the platform was removed from the pool. A probe trial was performed to verify the animal’s understanding of the platform location. The rat was released into the pool starting from one quadrant away from the target quadrant. The time spent by the rat in the target quadrant searching for the platform was recorded for 30 s^50^.

#### Dark/light test

The dark/light test is a common instrument for assessing anxiety. The apparatus consists of a simple chamber (50 cm × 100 cm × 40 cm) divided equally into a dark and a light compartment and the two compartments are connected by a small opening (7.5 cm × 8.5 cm)^51^. Rodents prefer darker areas over light areas; however, when presented in a novel environment, rodents tend to explore. These two conflicting emotions lead to observable anxiety-like symptoms. Without prior habituation or training, each rat was placed on the dark side and allowed to move freely between the two chambers for 1 min. The latency to emerge to the light side and the time spent in each compartment were recorded, and the percentage of time spent in the light was calculated to quantify performance in this test^52^.

### Rat sacrifice and sample collection

For sample collection, rats were humanely euthanized via intraperitoneal injection of an overdose of thiopental sodium (120 mg/kg body weight). Death was confirmed by absence of pedal reflexes, followed by bilateral thoracotomy, following MU-ACUC guidelines and AVMA 2020 recommendations^53,54^. For the biochemical assays, both hippocampi were extracted on a cold petri dish and snap-frozen immediately on dry ice to be stored at − 80 °C until analysis. The brains from the rats allocated for the histological study were preserved in 10% formalin overnight at 4 °C to ensure proper fixation of the brain tissue, which was then processed into paraffin blocks and stored at room temperature until the examination of the hippocampal tissues.

### Biochemical analysis

#### Inflammatory cytokine levels determination

Enzyme-linked immunosorbent assays (ELISAs) were used to detect the concentrations of the following inflammatory markers: Tumor necrosis factor alpha (TNF-α) (from Elabscience Biotechnology Co., Ltd., Wuhan, China, Cat NO: E-EL-R0019c), interleukin-6 (IL-6) (from the R&D System, MN, United States, Cat. No. R6000B) and interleukin 10 (IL-10) (from Wuhan, Fine Biotech Co., Wuhan, China, Cat. No. ER0033) in rat hippocampal samples according to the manufacturer’s protocols^55,56^.

#### Gene expression assessment

Relative mRNA expression of α-synuclein, calmodulin-1(CaM-1), Vit. D receptor (VDR) and caspase-3 were detected in brain tissues via real-time reverse transcription PCR (qRT‒PCR). During processing, liquid nitrogen was used to homogenize the hippocampal samples. To extract total cellular RNA, QIAzol (Qiagen, Germany) was used. A Thermo Scientific NanoDrop One (USA) was used to check the yield of RNA and determine its concentration and purity. The first strand of cDNA was synthesized from 1 µg of RNA via a Proflex Thermal Cycler (Applied Biosystems, USA) and a Sensi FASTTM cDNA Synthesis Kit (Bioline, UK). The primers were annealed for 10 min at 25 °C, reverse transcription was performed for 15 min at 42 °C, and inactivation was performed for 5 min at 85 °C. Azure Cielo 6 (Azure, USA) real-time PCR was used to amplify the cDNA templates. Ten microliters of SYBR Green PCR Master Mix (Bioline, UK), 1 µl of cDNA template, 2 µl (10 pmol/µl) of gene primer, and 7 µl of nuclease-free water made up the 20 µl reaction volume. After the thermal profile was adjusted for two minutes at 95 °C, 40 cycles were performed: five seconds of denaturation at 95 °C, followed by thirty seconds of annealing and extension at 60 °C. The CaM-1 primer was newly designed via Primer3, Version 4.1.0^57^, and the other primers were extracted from previous publications, as shown in Table 1. All primer specificity was checked via NCBI Primer-BLAST program^58^, and was purchased from Vivantis (Vivantis Technologies, Malaysia). Glyceraldehyde-3-phosphate dehydrogenase (GAPDH) was used as a reference gene. A melting curve analysis was performed to verify the specificity of the PCR products. The ΔΔCT method was used to calculate the fold change in gene expression and was computed in Microsoft^®^ Excel^®^ 2019 MSO (Version 2409 Build 16.0.18025.20160). The data are expressed as the fold change (2^−ΔΔCT^) relative to the gene expression of the control group^59^.

**Table 1:** Primers used for measurement of the relative mRNA expression of α-synuclein, calmodulin-1, vitamin D receptors and caspase-3.

Gene	Sequence	Reference/source
Caspase-3 (rat)	Forward ATGGAGAACAACAAAACCTCAGT	^60^
	Reverse TTGCTCCCATGTATGGTCTTTAC	
Vitamin D receptor (VDR) (rat)	Forward GCCCCTCATAAAGTTCCAGGTG	^61^
	Reverse GGATAGGCGGTCCTGAATGG	
α-Synuclein (rat)	Forward TGTCAAGAAGGACCAGATGGG	^62^
	Reverse TAGTCTTGGTAGCCTTCCTCT	
Calmodulin-1 (CaM-1) (rat)	Forward TGATAAAGATGGGGACGGCA	Primer3web version 4.1.0
	Reverse GCCATTGCCATCCTTGTCAA	

### Histological examination

#### H&E

Hippocampal tissues were used for histopathological examination. The formalin-fixed tissues were dehydrated with increasing concentrations of ethyl alcohol, cleared in xylene, and embedded in paraffin blocks until analysis. The tissues were dissected into serial 5 μm sections, prepared and stained with hematoxylin and eosin (H&E) for histopathological examination^63^.

#### Immunohistochemical investigation

The immunohistochemical staining procedures were performed as described by Farage et al. (2023). Hippocampal sections were dewaxed and immersed in a solution of 0.05 M citrate buffer, pH 6.8, for antigen retrieval. These sections were then treated with 0.3% H2O2 and blocked with protein. Then, the sections were incubated with a rat monoclonal Tau antibody (Invitrogen, MAS-12808, USA; 1:300 dilution). After they were rinsed with phosphate-buffered saline, they were incubated with a rodent monoclonal secondary antibody (Cat# K3468, EnVision+™ System Horseradish Peroxidase Labeled Polymer; Dako) for 30 min at room temperature. The slides were visualized with a DAB kit and subsequently stained with Mayer’s hematoxylin as a counterstain. The staining intensity was assessed in five randomly selected sample fields from each group by an expert pathologist who was blinded to the experimental groups, and the results are presented as a percentage of positive expression in a total of 500 neurons^64^.

#### Apoptotic cell counting

To count the average number of degenerated neurons within the hippocampus, H&E-stained sections from the brain hippocampus were used to quantify apoptotic cells, which were recognized as rounded or oval masses with dark eosinophilic cytoplasm and dense shrunken pyknotic nuclei^65^. An expert pathologist who was blinded to the experimental groups counted the number of apoptotic cells per high-power (x1000) field in three randomly selected sample fields from each group, and the percentages of degenerated neurons within 500 neuronal cells of the granular layer were calculated.

### Statistical analysis

SPSS Statistics for Windows (version 25; IBM Corp., Armonk, N.Y., USA), GraphPad Prism version 8.0.0 for Windows (GraphPad Software, San Diego, California, USA), and Microsoft^®^ Excel^®^ 2019 MSO (Version 2409 Build 16.0.18025.20160) were used for the computing, analysis and presentation of the data. The data were checked for normality via the Shapiro–Wilk test. Normally distributed data are presented as the mean ± SEM and were analyzed by one-way ANOVA followed by the post hoc Tukey test. For nonnormally distributed data, the nonparametric Kruskal‒Wallis test followed by post hoc pairwise comparisons with Dunnett correction was used, and the data are presented as the median (IQR). A P value less than 0.05 was considered significant.

## Results

### Behavioral tests

#### Open field test

**Fig. 1:**
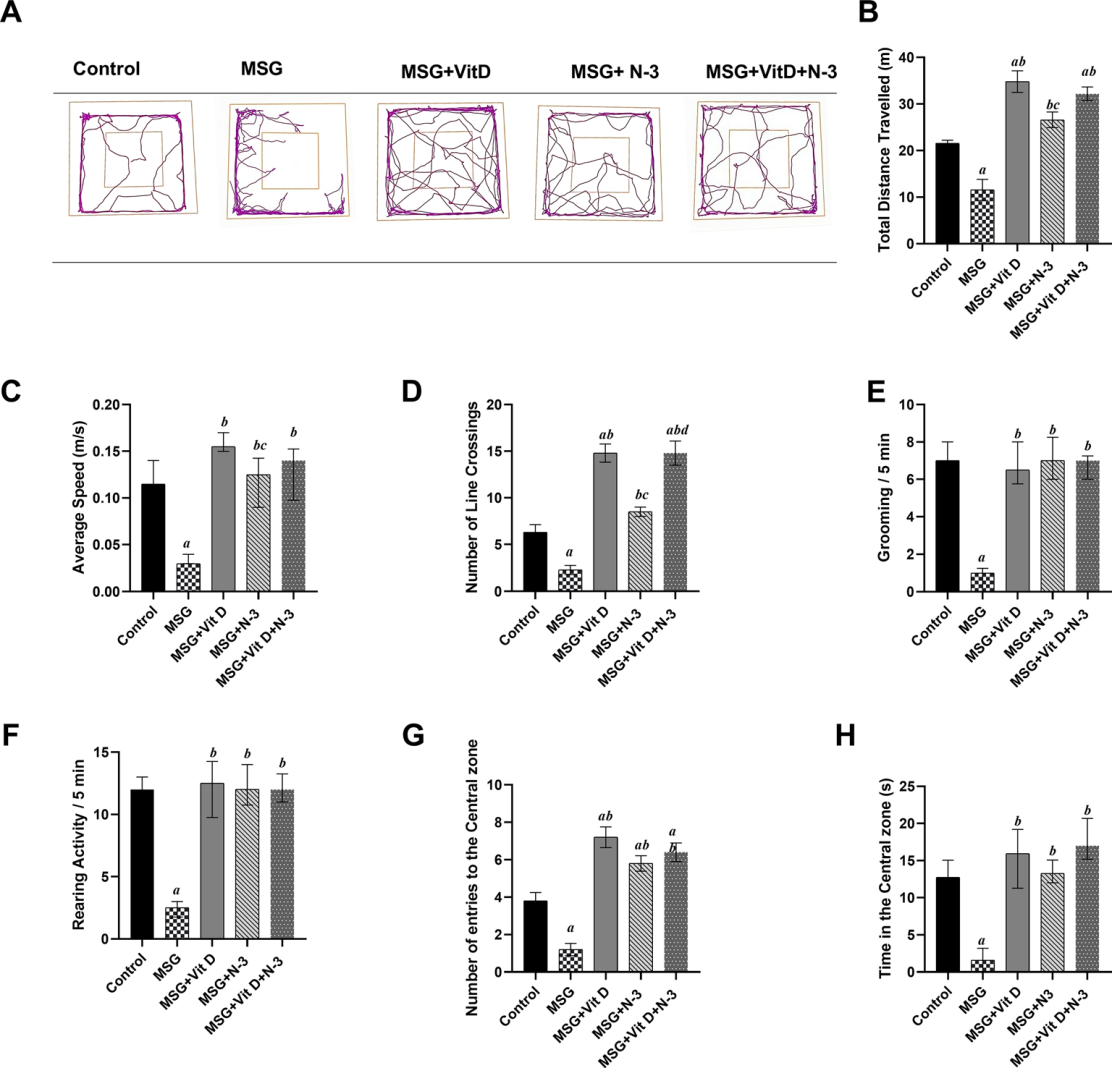
Rats’ performances in the open field test. The track plots show the positions of the animals during the entire duration of the test (**A**); the inner squares denote the supposed central zone. The graphs show the total distance traveled by the animals (**B**), average speed of animal movement (**C**), number of line crossings during the test period (**D**), number of grooming behaviors (**E**), number of rearing activities (**F**), number of entries into the central zone (**G**) and time spent in the central zone (**H**) among the experimental groups; the control group. MSG (Monosodium glutamate), Vit D (vitamin D), N-3 PUFAs (omega-3 polyunsaturated fatty acids) and the combined treatment group (MSG + Vit D + N-3). The data are expressed as the means ± SEMs or medians (IQRs) (*n* = 10 per group). Symbols above individual bars indicate a significant P value of < 0.05 for multiple pairwise comparisons between groups according to Tukey’s post hoc HSD test or Dunn’s multiple comparison test; (a): for significance vs. control; (b): for significance vs. MSG; (c): for significance vs. Vit D; and (d): for significance vs. N-3 PUFAs.

Statistical analyses of rats’ behaviors in the open field arena at 4 weeks post-treatment have demonstrated robust treatment effects across all behavioral parameters. Parametric one-way ANOVA revealed significant group differences in number of line crossings (*F*(4,45) = 39.20, *P* < 0.001), total distance traveled (*F*(4,45) = 27.00, *P* < 0.001), and number of entries to the central zone (*F*(4,45) = 28.18, *P* < 0.001). Non-parametric Kruskal-Wallis tests further corroborated these effects for average speed (*H*(4) = 30.41, *P* < 0.001), time spent in the central zone (*H*(4) = 30.68, *P* < 0.001), grooming frequency (*H*(4) = 24.62, *P* < 0.001), and rearing activity (*H*(4) = 23.94, *P* < 0.001). Post-hoc pairwise comparisons confirmed that the MSG group moved significantly less and slower than the control group did, as indicated by the total distance traveled (*P* < 0.001) and the average speed in the open field arena (*P* < 0.01). On the other hand, the distance traveled by the Vit D- and/or N-3 PUFA-treated rats was significantly greater than that traveled by the MSG-treated rats (*P* < 0.001, *P* < 0.01) for the individual treatments respectively, and (*P* < 0.001) for the combination therapy. Notably, the rats treated with Vit D traveled much farther than those treated with N-3 PUFAs (*P* < 0.01). Additionally, the rats treated with Vit D or combination treatment scored longer distances than the control rats (*P* < 0.001), but there was no significant difference between the individual treatment with N-3 PUFAs or Vit D and the combination therapy (*P* > 0.05). Similarly, compared with those in the MSG group, the rats gained significantly faster speeds of movement upon treatment with Vit D (*P* < 0.001), N-3 PUFAs (*P* < 0.01), or the combined treatment (*P* < 0.001). Compared with those in the N-3 PUFA group, significantly greater speeds were achieved in the Vit-D-treated group (*P* < 0.05), whereas there was no significant speed difference between the individual treatments with N-3 PUFAs or Vit D and the combination therapy group or the control group (*P* > 0.05) (Fig. 1B, C).

Compared with normal control rats, the number of crossed fields was significantly lower following MSG administration (*P* < 0.01). Compared with the MSG group (*P* < 0.001), the Vit D- and/or N-3 PUFA-treated group presented a significant increase in crossings in the open field. Notably, rats treated with Vit D or combination therapy presented a greater number of crossings than did those treated with N-3 PUFAs or even normal control rats (*P* < 0.001) for both (Fig. 1D).

The number of grooming activities in the open field was significantly lower after MSG administration during the observation period compared to intact control rats (*P* < 0.001), whereas rats treated with Vit D and/or N-3 PUFAs presented significantly greater grooming activities and reached the normal scores recorded in the control groups (*P* < 0.01, *P* < 0.001) for individual treatments and (*P* < 0.001) for combined treatments compared with those in the MSG group. There was no significant difference between the individual treatments (N-3 PUFAs vs. Vit D) (*P* > 0.05) or between the separate treatments and the combination therapy (*P* > 0.05) (Fig. 1E).

Similarly, fewer rearing behaviors were recorded in the MSG rats than in the control rats (*P* < 0.001). Unlike MSG-treated rats, the rearing behavior of rats that received Vit D and/or N-3 PUFAs was significantly greater, matching that of the control group (*P* < 0.001) for both the individual and combined treatments. There was no significant difference between the individual treatments (N-3 PUFAs vs. Vit D) (*P* > 0.05) or between the separate treatments and the combination therapy (*P* > 0.05) (Fig. 1F).

The observation of other anxiety-like behavioral activity in the open field test revealed that the MSG group tended to visit the central zone less often than the control group did, as indicated by the number of crossings (*P* < 0.001) and time spent there (*P* < 0.05). Vit D- and/or N-3 PUFA-treated animals entered more time into the central zone than MSG-treated rats did (*P* < 0.001) for the individual and combined treatments. Additionally, the zone entry scores of the treatment groups were significantly greater than those of the control group (*P* < 0.001) for Vit D, (*P* < 0.05) for N-3 PUFAs, and (*P* < 0.001) for the combined treatment. There was no significant difference between the individual treatments (N-3 PUFAs vs. Vit D) or between the separate treatments and the combination therapy (*P* > 0.05). Moreover, Vit D- and/or N-3 PUFA-treated rats had scored the normal time recorded by the control rats in the central zone, as they spent much more time in the central zone than MSG-treated rats did; (*P* < 0.001) and (*P* < 0.05) for individual treatments, respectively, and (*P* < 0.001) for combined treatment. Notably, rats that received the combination therapy stayed in the central zone longer than those treated with N-3 PUFAs only (*P* < 0.01), but there was no significant difference between the individual treatments (N-3 PUFAs vs. Vit D) (*P* > 0.05) (Fig. 1G, H).

#### Novel object recognition test

**Fig. 2:**
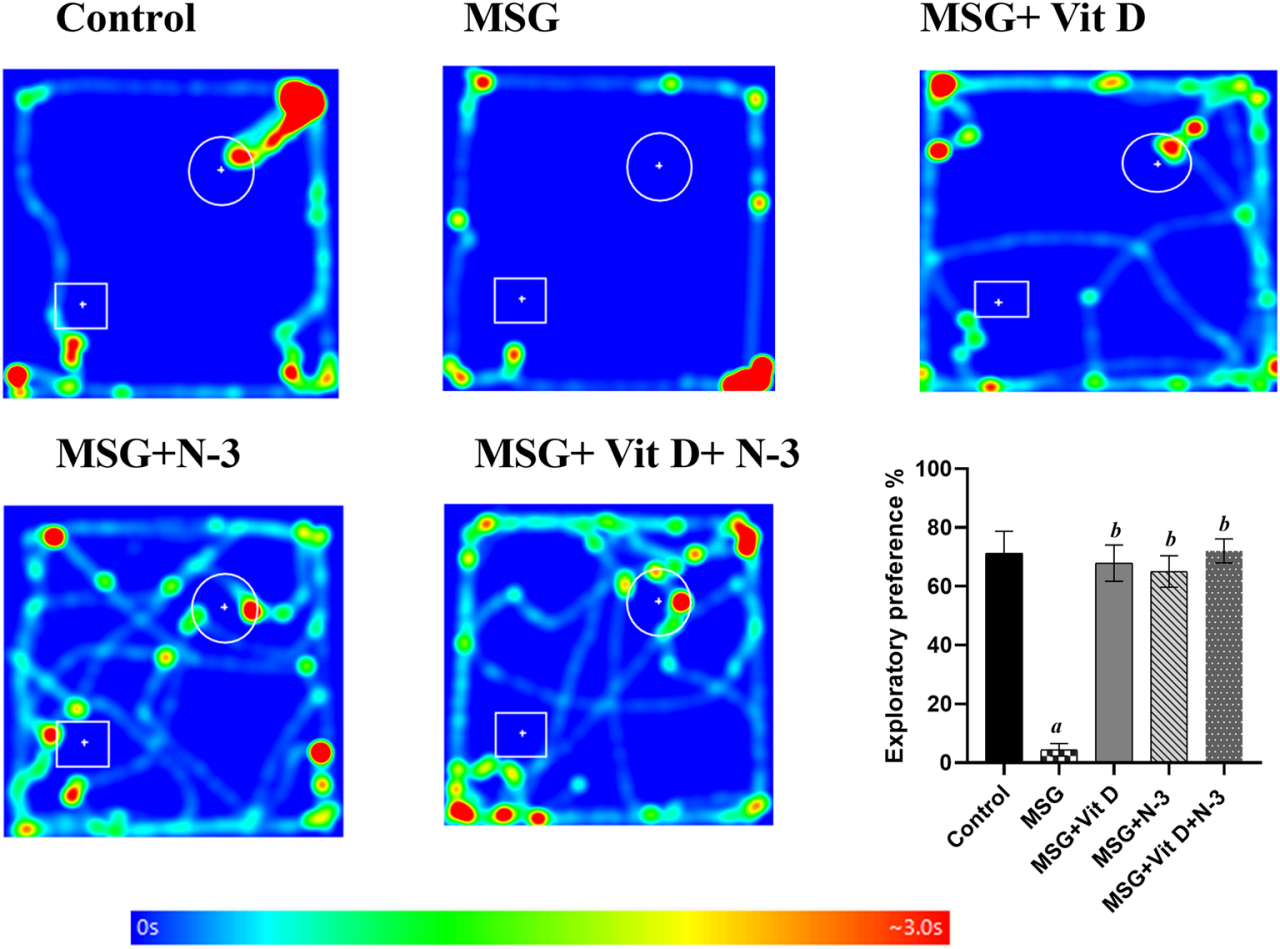
Rats’ exploratory performances in the novel object recognition test. The heatmaps represent the positions of the rats throughout the test duration; blue represents the shortest time, and red represents the longest time. The circle denotes the site of the novel object, whereas the rectangle represents the familiar object. The graph shows the exploratory preference behavior among the experimental groups: MSG (Monosodium glutamate), Vit D (vitamin D), N-3 PUFAs (omega-3 polyunsaturated fatty acids), and the combined treatment group (MSG + Vit D + N-3). Data are expressed as the median (IQR) (*n* = 10 per group). Symbols above individual bars indicate a significant P value of < 0.05 for multiple pairwise comparisons between groups according to Dunn’s multiple comparison test; (a): for significance vs. control; (b): for significance vs. MSG; (c): for significance vs. Vit D; and (d): for significance vs. N-3 PUFAs.

For NOR performance, significant disparities between groups in exploratory preference were identified using Kruskal-Wallis analysis (*H(4) = 17.13*, *P* < 0.01). Rats treated with MSG tended to huddle in the corners of the arena and spent much less time investigating the familiar and novel objects than intact control rats did (*P* < 0.01) according to the post-hoc tests, whereas Vit D and/or N-3 PUFA-treated rats spent the same amount of time investigating the familiar and novel objects as normal rats did in the control group, showing greatly prolonged exploration time for both objects and increased exploratory preference for the novel objects compared with those of rats treated with MSG (*P* < 0.01, *P* < 0.05) for individual treatments and (*P* < 0.01) for combined treatment. There was no significant difference between the individual treatments (N-3 PUFAs vs. Vit D) or between the separate treatments and the combination therapy (*P* > 0.05) (Fig. 2).

#### T-maze test

**Fig. 3:**
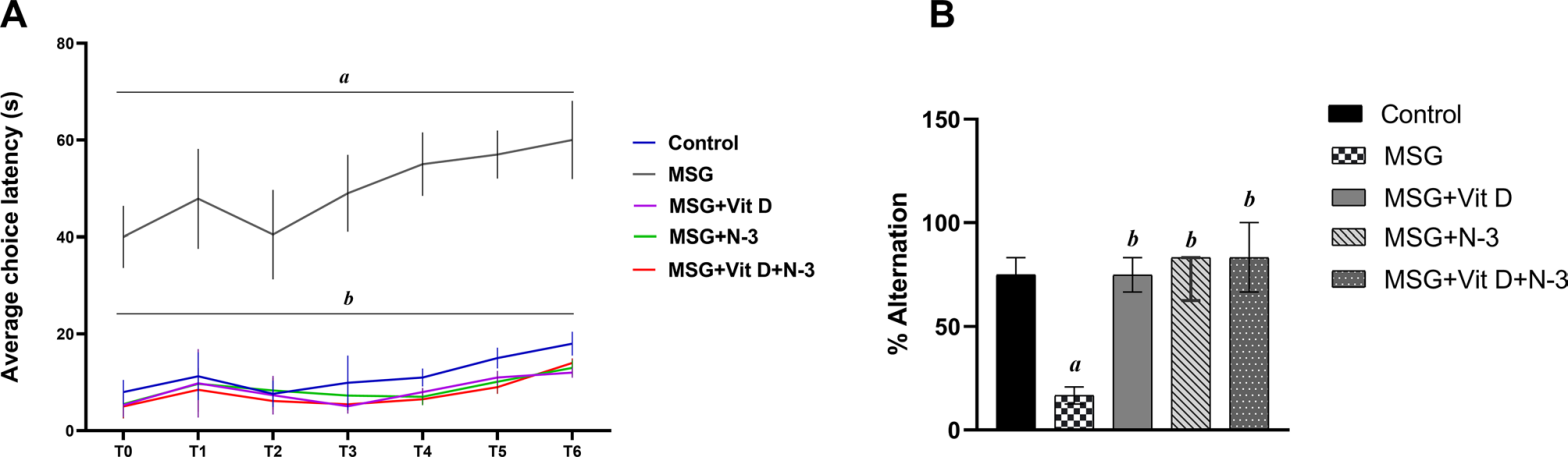
Spatial working memory performance in the T-maze. Graphs show the average choice latency across trials (**A**) and the percent of correct alternations among the experimental groups (**B**); the control group, MSG (Monosodium Glutamate), Vit D (vitamin D), N-3 PUFAs (omega-3 polyunsaturated fatty acids) and the combined treatment group (MSG + Vit D + N-3). Data are expressed as the median (IQR) (*n* = 10 per group). Symbols above individual bars indicate a significant P value of < 0.05 for multiple pairwise comparisons between groups according to Dunn’s multiple comparison test; (a): for significance vs. control; (b): for significance vs. MSG; (c): for significance vs. Vit D; and (d): for significance vs. N-3 PUFAs.

The performance in the T-maze revealed that both the percentage of correct alternations (*H(4) = 25.77*, *P* < 0.001) and the latencies to enter cross arms (*H(4) = 19.69*, *P* < 0.001) had significant differences across groups, according to Kruskal-Wallis analysis. Post-hoc analysis demonstrated a significantly greater delay of the MSG-treated rats before entering a cross arm (*P* < 0.001), in addition to a decreased tendency toward the correct alternations (*P* < 0.01) compared to the control group. Upon treatment, the performance of rats in the T-maze almost matched that of control rats; that is, a significantly shorter latency to enter the arms was observed in response to Vit D and/or N-3 PUFA treatment than in the MSG group (*P* < 0.01) for individual treatments and (*P* < 0.001) for combined treatment. Additionally, significantly greater percentages of correct alternations were recorded in the treatment groups than in the MSG group (*P* < 0.01) for Vit D, (*P* < 0.001) for N-3 PUFAs, and (*P* < 0.001) in the combined treatment group. There was no significant difference between the effects of the individual treatments (N-3 PUFAs vs. Vit D) on the T-maze measurements (*P* > 0.05). Similarly, there was no significant difference between the individual treatment with N-3 PUFAs or Vit D and the combined treatment in terms of latency (*P* > 0.05) or percentage of correct alternations (*P* > 0.05) (Fig. 3).

#### Morris water maze test

**Fig. 4:**
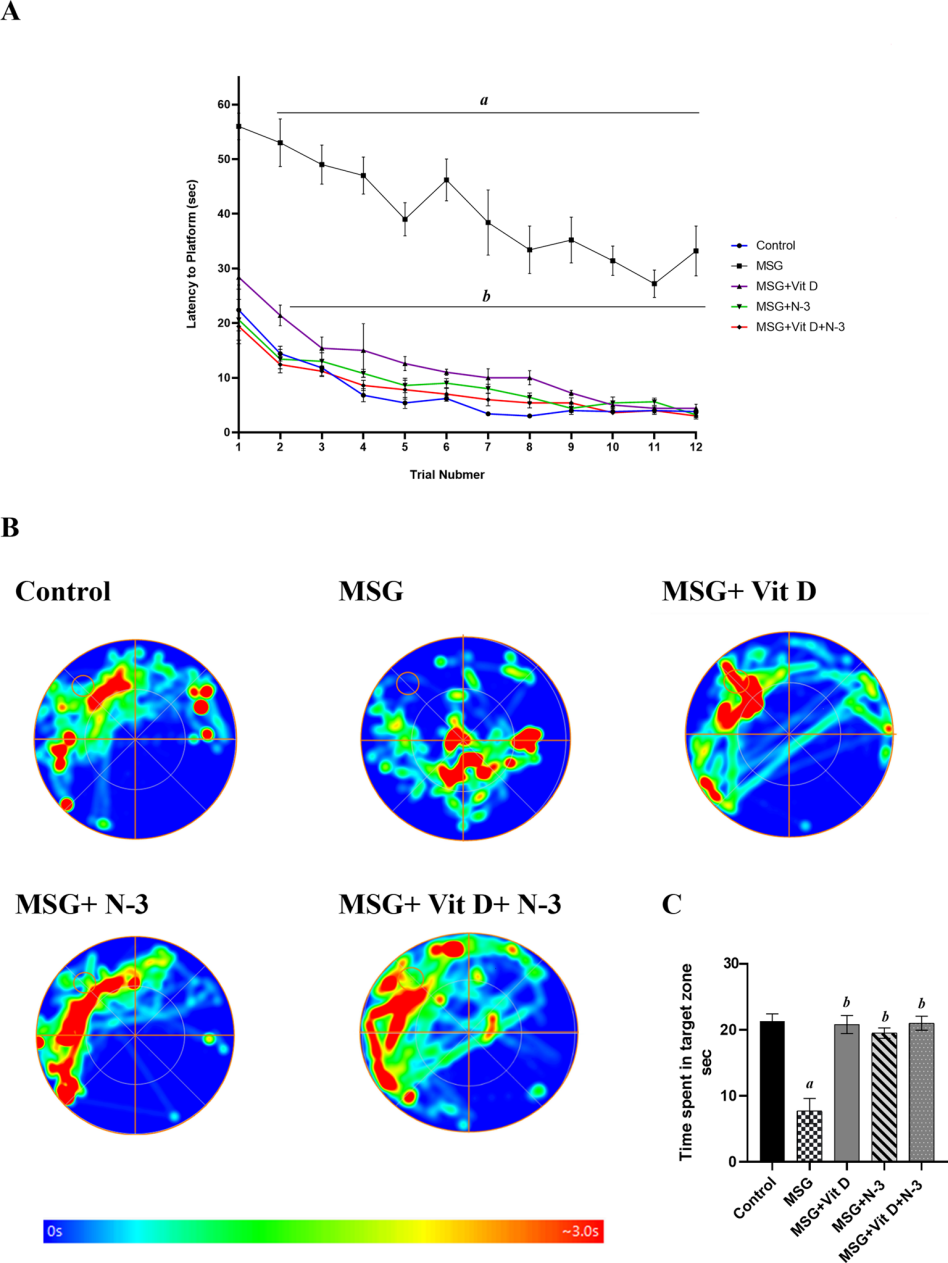
Learning performances and spatial working memory in the water maze. The learning curves of the escape latencies to reach the platform during the 12 training trials (**A**) and heatmap representation of the time spent by the animals in different parts of the apparatus during the probe test (**B**), with blue as the shortest time and red as the longest. The circles denote the supposed location of the removed platform in the target zone. The graph shows the average time spent in the target zone among the experimental groups during the probe test (**C**), the control group, MSG (Monosodium glutamate), Vit D (vitamin D), N-3 PUFAs (omega-3 polyunsaturated fatty acids), and the combined treatment group (MSG + Vit D + N-3). Data are expressed as the median (IQR) (*n* = 10 per group). Symbols above individual bars indicate a significant P value of < 0.05 for multiple pairwise comparisons between groups according to Dunn’s multiple comparison test; (a): for significance vs. control; (b): for significance vs. MSG; (c): for significance vs. Vit D; and (d): for significance vs. N-3 PUFAs.

According to Kruskal-Wallis analysis, there were notable variations between the groups in both latencies to reach the platform (*H(4) = 31.92*, *P* < 0.001) during the training trials and the time spent in the target zone during the probe trial (*H(4) = 12.34*, *P* < 0.05). Post-hoc tests demonstrated that MSG group rats presented impaired learning performance in the MWM in all training trials compared with normal control rats, as demonstrated by a significantly longer latency time to find the hidden platform (*P* < 0.001) and failure to achieve the normal learning curve. On the other hand, rats treated with either N-3 PUFAs, Vit D, or combination therapy were able to locate the hidden platform more rapidly than MSG rats were (*P* < 0.001, *P* < 0.01,) for separate treatment respectively and (*P* < 0.001) for the combined therapy. In addition, latencies declined progressively in the successive swims, showing suspected learning curves similar to those of the control group. There was no significant difference between the individual treatments (N-3 PUFAs vs. Vit D) (*P* > 0.05) or between the separate treatments and the combination therapy (*P* > 0.05) (Fig. 4A).

Spatial reference memory analysis in the probe test of the MWM revealed that the group was significantly confused in all the maze quadrants and spent less time in the target (platform) zone than the control group did; the memory retention (*P* < 0.01) was decreased, whereas the memory functions were significantly improved after Vit D and/or N-3 PUFA treatment, which was almost comparable to the results observed in the normal control group and was evidenced by the significantly longer swimming time in that target quadrant than in the MSG group (*P* < 0.01) for both the individual and combined treatments. There was no significant difference between the individual treatments (N-3 PUFAs vs. Vit D) or between the separate treatments and the combination therapy (*P* > 0.05) (Fig. 4B, C).

#### Dark/light test

**Fig. 5:**
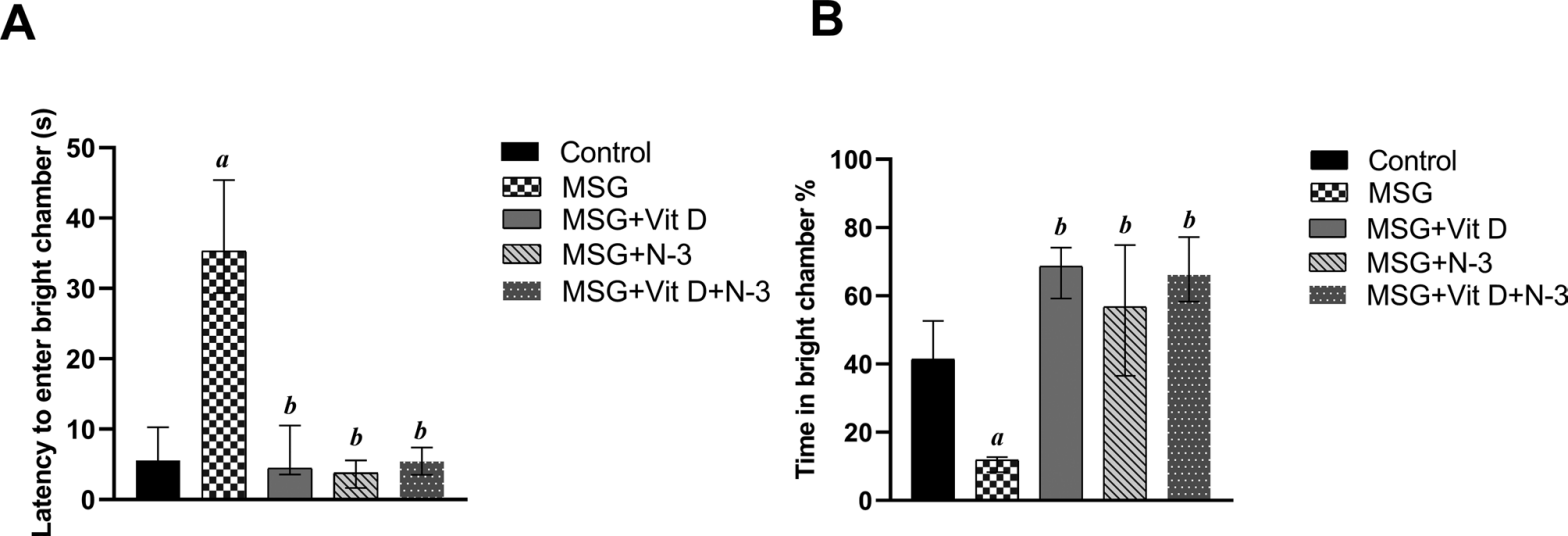
Rats’ performances in the dark‒light box. The graphs show the latency to the 1st cross to the light side (**A**) and the percentage of time spent in the light chamber (**B**) among the experimental groups: the control group, MSG (monosodium glutamate), Vit D (vitamin D), N-3 PUFAs (omega-3 polyunsaturated fatty acids) and the combined treatment group (MSG + Vit D + N-3). Data are expressed as the median (IQR) (*n* = 10 per group). Data are expressed as the median (IQR). Symbols above individual bars indicate a significant P value of < 0.05 for multiple pairwise comparisons between groups according to Dunn’s multiple comparison test; (a): for significance vs. control; (b): for significance vs. MSG; (c): for significance vs. Vit D; and (d): for significance vs. N-3 PUFAs.

During the dark/light test, significant differences in both latencies to enter light across groups *(H(4) = 26.18*, *P* < 0.001) and % of time spent in the light chamber *(H(4) = 30.05*, *P* < 0.001) as revealed by the Kruskal-Wallis test. Post-hoc analysis demonstrated that the MSG-exposed group exhibited a significantly greater latency before crossing to the light chamber in comparison to the control group (*P* < 0.01). Likewise, rats in the MSG-treated group spent much less time on the light side than did the control rats (*P* < 0.001). Compared with MSG alone, treatment with Vit D and/or N-3 PUFAs appeared to decrease these anxiety-related behaviors, consistent with normal performance, as evidenced by markedly decreased latency before the rats crossed the light chamber (*P* < 0.01) for Vit D, (*P* < 0.001) for N-3 PUFAs and the combined treatment. Similarly, the treated rats spent a significantly greater percentage of time on the light side than the MSG-treated rats did (*P* < 0.001) for individual and the combined treatment. There was no significant difference between individual treatments (N-3 PUFAs vs. Vit D) for both the dark/light measurements (*P* > 0.05) or between the separate treatments and the combination therapy in terms of latencies to enter (*P* > 0.05) or the time spent on the bright side (*P* > 0.05) (Fig. 5).

### Pathological analysis of hippocampal tissue damage induced by MSG

#### Histological examination

**Fig. 6:**
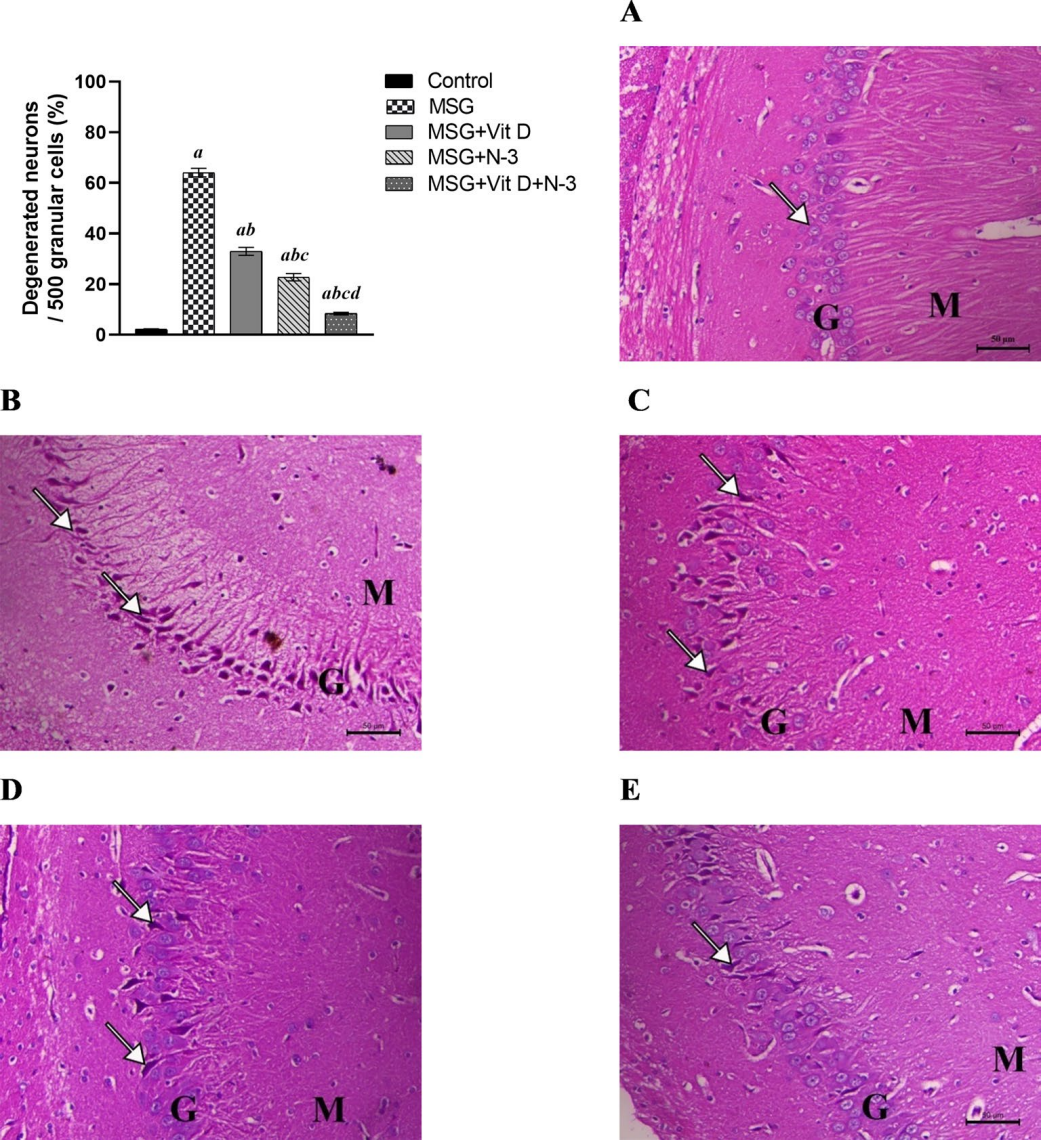
Histological findings in the hippocampal tissues of the experimental groups. The arrows indicate ischemic neuronal injury to the granular layer (the G and M letters indicate the granular and molecular layers, respectively), H&E, X200, scale bar = 50 μm. The bar graph represents the percentage of degenerated neurons/500 cells within the hippocampal granular layer among the experimental groups; (**A**) (the control group), (**B**) (monosodium glutamate group; MSG), (**C**) (vitamin D group; MSG + Vit D), (**D**) (omega-3 polyunsaturated fatty acid group; MSG + N-3), and (**E**) (the combined treatment group (MSG + Vit D + N-3)). The data are expressed as the means ± SEMs (*n* = 3 per group). Symbols above individual bars indicate a significant P value at < 0.05 for multiple pairwise comparisons between groups according to the Tukey HSD post hoc test; (a): for significance vs. control; (b): for significance vs. MSG; (c): for significance vs. Vit D; and (d): for significance vs. N-3 PUFAs.

Examination of H&E sections of the hippocampi of the rats in the control groups revealed a normal cell structure and arrangement, with normal neuronal cells in the granular cell layer and normal nerve fibers in the molecular layers. MSG-treated rats presented a hippocampus with shrunken and degenerated neurons with pyknotic nuclei and increased perineural spaces. Additionally, severe ischemic neuronal degeneration associated with atrophy, loss of cytoplasmic and nuclear architectures, consequent atrophy of the granular layer, and rarefaction of the molecular layer were observed. Hippocampi from N-3 PUFA- and/or Vit D-treated rats presented a marked decrease in the features of ischemic neuronal injury to the hippocampal granular layer. These findings were confirmed via statistical analysis of the percentage of degenerated neurons per 500 cells within the hippocampus, where one-way ANOVA demonstrated a highly significant effect on apoptotic cell counts across experimental groups (*F(4*, 10) = 380.1, *P* < 0.001), This exceptionally large F-statistic underscores the pronounced treatment-induced modulation of apoptotic mechanisms between groups. In line with that, there was a significant increase in the mean number of degenerated cells in all areas in the MSG group compared with those in the control group (*P* < 0.001). Conversely, rats treated with Vit D and/or N-3 PUFAs presented a considerable decrease in the mean number of degenerated neural cells compared with those in the MSG group (*P* < 0.001) for both the individual and combined treatments. Notably, rats treated with N-3 PUFAs had significantly fewer degenerated neural cells in their hippocampi than rats treated with Vit D alone (*P* < 0.01). Compared with the separate N-3 PUFA or Vit D treatments, the combination therapy resulted in fewer degenerated neurons within the hippocampus (*P* < 0.001). However, the neural damage shown in all three treatment groups was still significantly greater than that in the control group (*P* < 0.001) for both the individual treatments and the combined therapy (*P* < 0.05) (Fig. 6).

#### Tau protein accumulation in hippocampal neurons

**Fig. 7:**
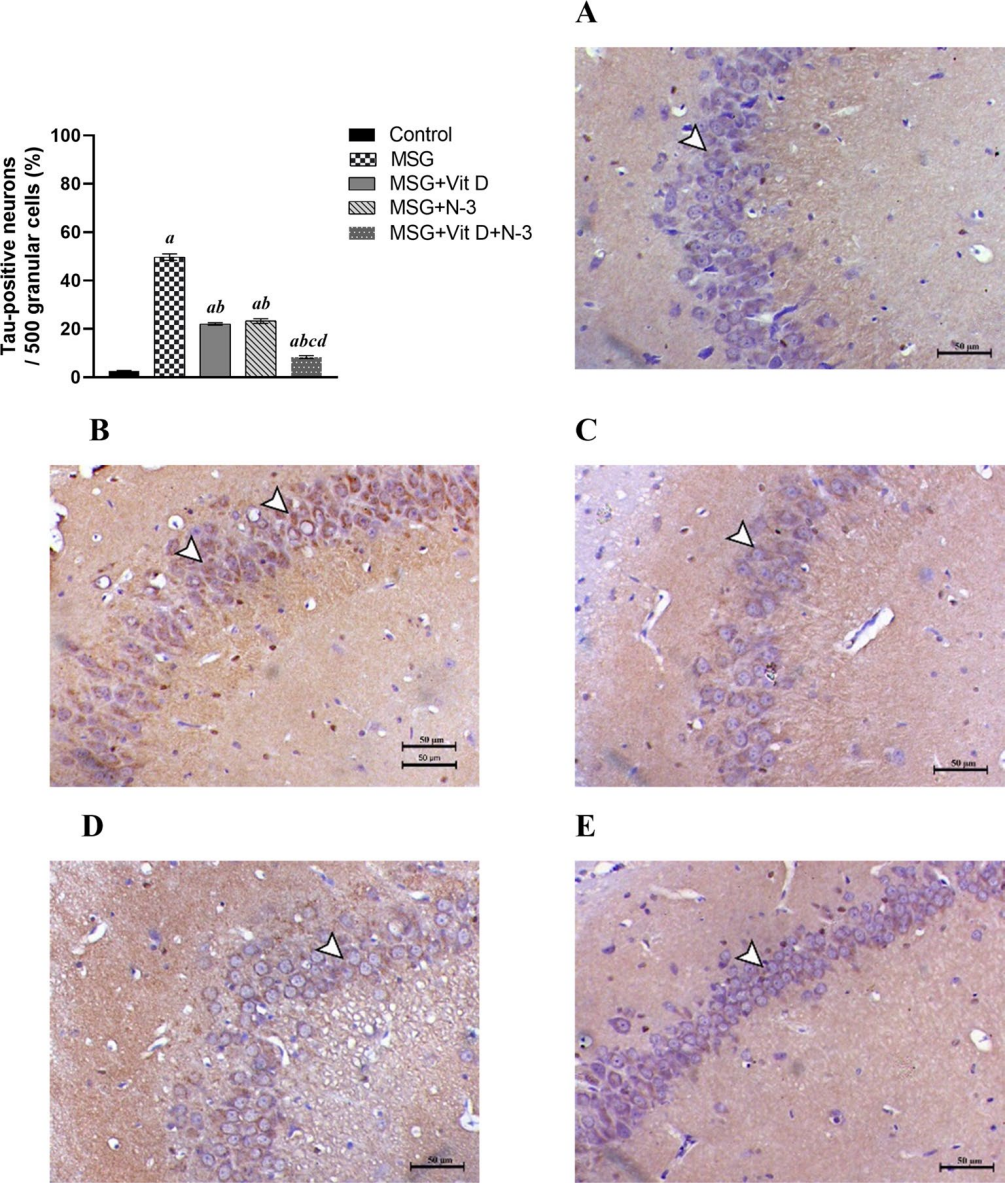
Tau immunostaining results in the hippocampi of the experimental groups. Arrowheads refer to tau-positive immunostained neurons within the granular cell layer of the hippocampus, Tau IHC, X200, bar = 50 μm; (**A**) (the control group), (**B**) (monosodium glutamate group; MSG), (**C**) (vitamin D group; MSG + Vit D), (**D**) (omega-3 polyunsaturated fatty acid group; MSG + N-3), and (**E**) (the combined treatment group (MSG + Vit D + N-3)). The data are expressed as the means ± SEMs (*n* = 5 per group). Symbols above individual bars indicate a significant P value of < 0.05 for multiple pairwise comparisons between groups according to the Tukey HSD post hoc test; (a): for significance vs. control; (b): for significance vs. MSG; (c): for significance vs. Vit D; and (d): for significance vs. N-3 PUFAs.

Tau-immunostained sections of hippocampal tissues from the control group presented few immunopositive brownish cells. MSG-treated rats presented a marked increase in tau immunostaining within the neuronal cells of the hippocampal granular cells. Sections from rats treated with N-3 PUFAs and/or Vit D revealed a remarkable decrease in tau antibody deposition within the neuronal cells of the granular and molecular layers. The results were verified by statistical analysis based on the percentage of positive immune-stained neurons within 500 cells in the hippocampi of the experimental groups. One-way ANOVA identified a profound intergroup difference in tau accumulation (*F(4*, 20) = 493.8, *P* < 0.001). This robust statistical outcome underscores the substantial impact of the interventions on modulating tau-related neurodegenerative pathways. In addition, compared with that in control rats, the percentage of hippocampal tau-positive neurons in MSG-treated rats was significantly greater (*P* < 0.001). Compared with the MSG-treated group, the Vit D- and/or N-3 PUFA-treated groups presented a significant reduction in the number of tau-positive neurons (*P* < 0.001); however, there was no significant difference between the effects of either individual treatment (N-3 PUFAs vs. Vit D) (*P* > 0.05). Notably, rats that received the combined treatment presented a greater reduction in tau staining in their hippocampi than did those treated with Vit D or N-3 PUFAs alone (*P* < 0.001). However, tau deposition in all three treatment groups was significantly greater than that in the control group (*P* < 0.001) for both separate treatments and (*P* < 0.001) for the combined therapy (Fig. 7).

### Biochemical analysis

#### Concentration of inflammation-related markers

**Fig. 8:**
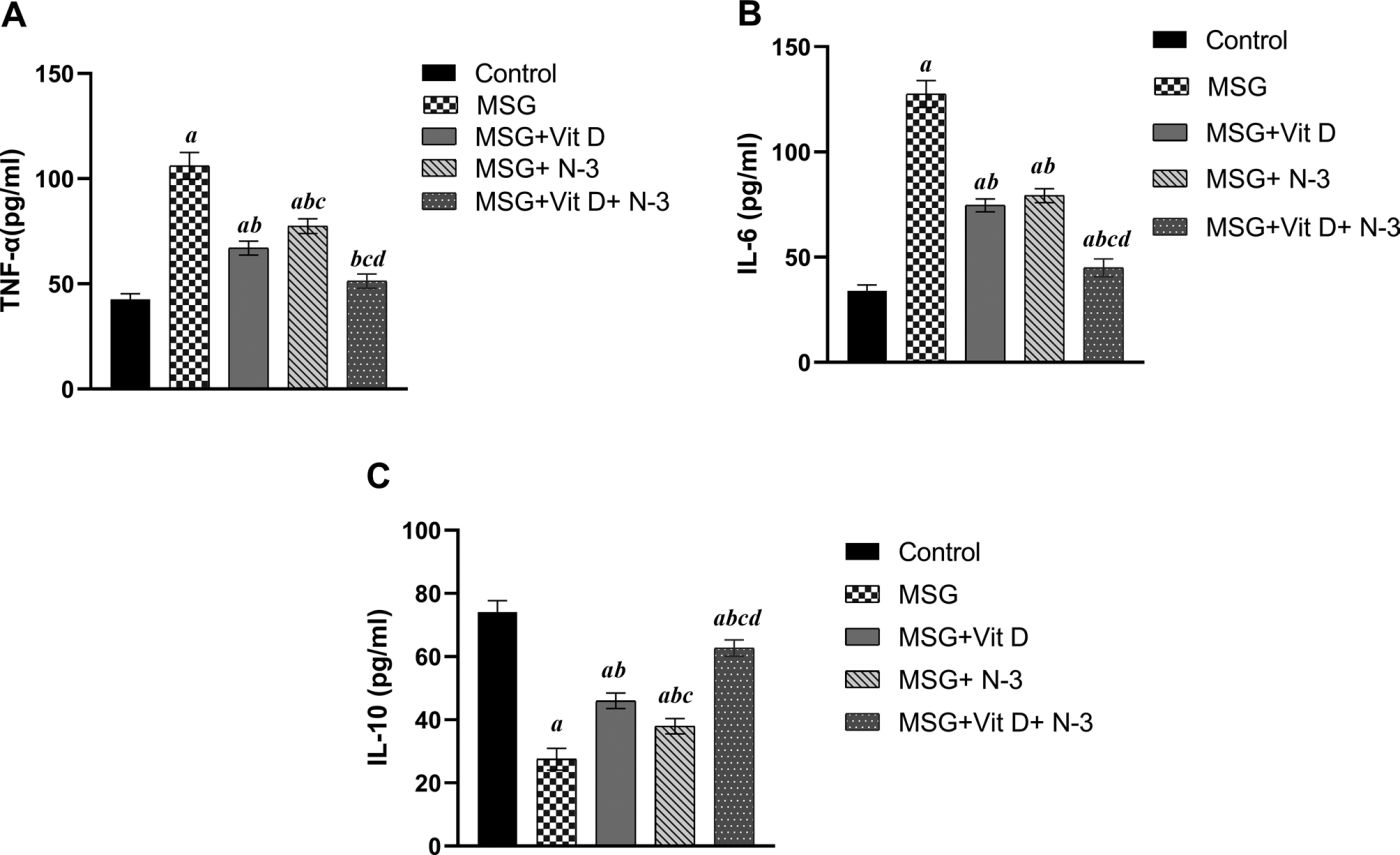
Hippocampal concentrations of inflammation-related cytokines. The graphs show the ELIZA results for the hippocampi of the rats in the following groups: (**A**): tumor necrosis factor-alpha (TNF-α), (**B**): interleukin-6 (IL-6), and (**C**) interleukin-10 (IL-10) in the brain hippocampi of the experimental groups; control, monosodium glutamate group; (MSG + vitamin D group; (MSG + Vit D), omega-3 polyunsaturated fatty acid group; (MSG + N-3), and the combined treatment group; (MSG + Vit D and N-3). The data are expressed as the means ± SEMs. Symbols above individual bars indicate a significant P value < 0.05 for multiple pairwise comparisons between groups by Tukey’s HSD post hoc test; (a): for significance vs. control; (b): for significance vs. MSG; (c): for significance vs. Vit D; and (d): for significance vs. N-3 PUFAs.

The inflammatory marker levels in the hippocampus after MSG exposure in the presence or absence of Vit D or N-3 PUFA treatment are presented in Fig. 8. One-way ANOVA revealed profound effect of the treatments on TNF-α (*F (4*,*10) = 112.3*, *P* < 0.001) and IL-6 (*F (4*,*10) = 226.5*, *P* < 0.001) levels across groups. Post-hoc analysis showed that the levels of TNF-α and IL-6 were significantly greater in the hippocampal tissues of the MSG-treated group than in those of the normal control group (*P* < 0.001). Compared with the MSG-treated group, the N-3 PUFA and/or Vit D treatment groups presented significantly lower cytokine levels (*P* < 0.001) in the individual and combined treatment groups. There was no significant difference in the effect of either individual treatment (N-3 PUFAs vs. Vit D) on the IL-6 level (*P* > 0.05); however, the TNF-α level was significantly lower in the rats treated with Vit D or the combination therapy than in those treated with individual N-3 PUFAs (*P* < 0.001) in both groups; nevertheless, the levels of these inflammatory cytokines in the different treatment groups were significantly greater than those in the control group (*P* < 0.001). Notably, the hippocampi of the rats treated with the combination therapy showed a more considerable attenuation of TNF-α (*P* < 0.001) and IL-6 levels (*P* < 0.001) compared with those of the separate treatments with Vit D or N-3 PUFAs. Although combined treatment was able to decrease TNF-α back to its normal level recorded in the control group (*P* < 0.05), the rats that received combined therapy still had significantly greater IL-6 levels than the control rats did (*P* < 0.01) (Fig. 8A, B).

On the other hand, IL-10 concentrations exhibited statistically significant variation across experimental groups, as determined by one-way ANOVA (F(4, 10) = 121.7, *P* < 0.001). The administration of MSG significantly depleted the IL-10 levels in the MSG-treated rats compared with those in the normal control rats (*P* < 0.001). In contrast to those in the MSG group, the IL-10 levels significantly increased in both the N-3 PUFA (*P* < 0.01) and Vit D treatment and combination therapy (*P* < 0.001) groups; however, the high IL-10 levels recorded in all three treatment groups were still significantly lower than those recorded in the control group (*P* < 0.001) for both individual treatments and (*P* < 0.01) for the combined treatment. Notably, the increase in Vit D-associated IL-10 was significantly greater than in N-3 PUFAs (*P* < 0.05). Similarly, rats treated with the combination therapy presented significantly greater IL-10 levels in their hippocampi than those treated with Vit D or N-3 PUFAs alone (*P* < 0.001) for both (Fig. 8C).

#### Brain hippocampus mRNA expression

**Fig. 9:**
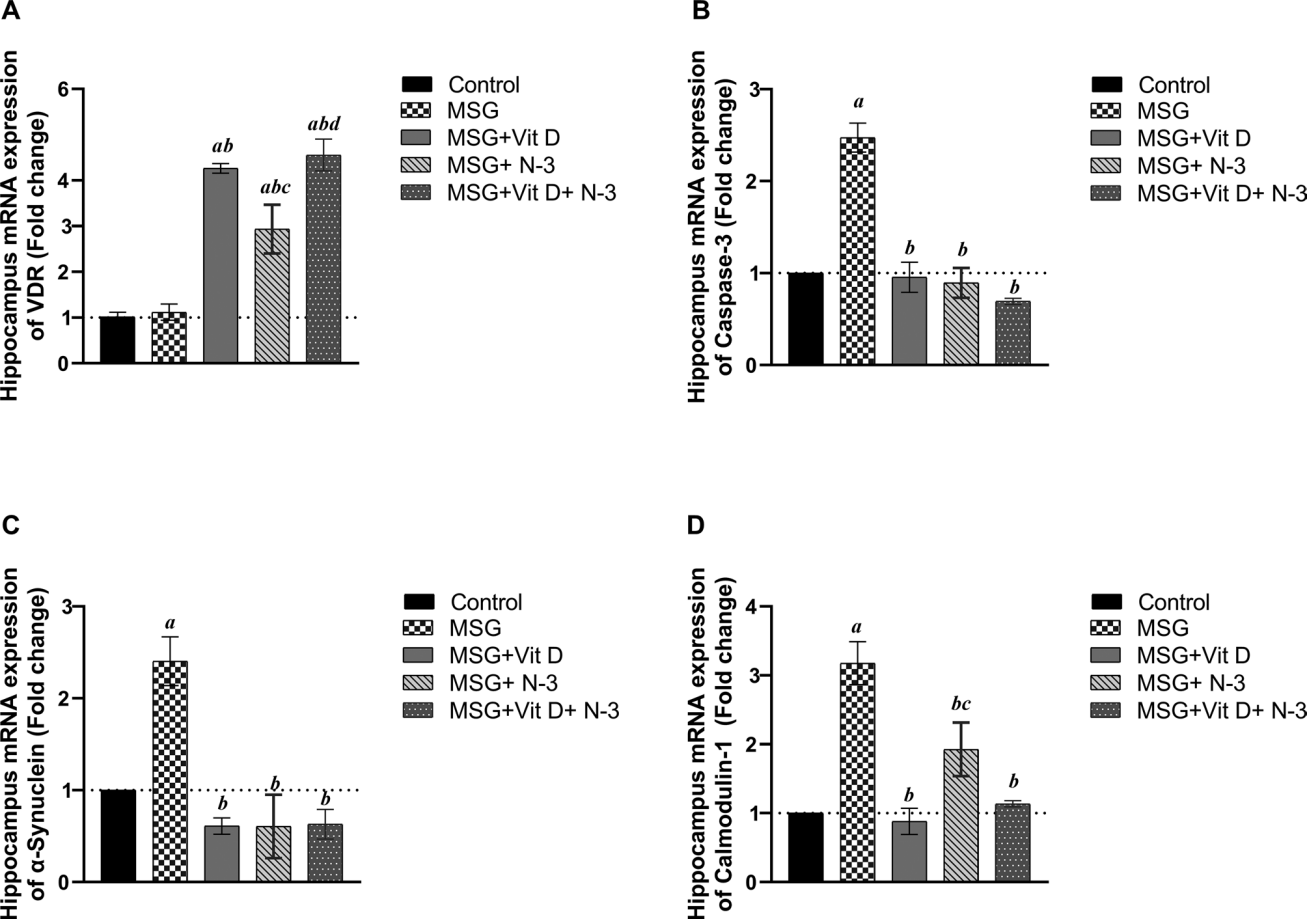
Representation of changes in rat hippocampus mRNA expression. The graphs show the fold changes in the expression of VDR (**A**), caspase-3 (**B**), α-synuclein (**C**), and calmodulin-1 (CaM-1) (**D**) in the brain hippocampi of the experimental groups relative to the control group. Monosodium glutamate group; (MSG), vitamin D group; (MSG + vitamin D), omega-3 polyunsaturated fatty acid group; (MSG + N-3), combined treatment group; (MSG + vitamin D and N-3). The data are expressed as the means ± SEMs (*n* = 10 per group). Symbols above individual bars indicate a significant P value of < 0.05 for multiple pairwise comparisons between groups according to the Tukey HSD post hoc test; (a): for significance vs. control; (b): for significance vs. MSG; (c): for significance vs. Vit D; and (d): for significance vs. N-3 PUFAs.

As shown in Fig. 9B–D, at 4 weeks post-treatment, Vit D and/or N-3 PUFAs were able to alter hippocampal gene expression substantially. One-way ANOVA revealed statistically significant differences in the expression of caspase-3 (*F(4*,*10) = 32.97*, *P* < 0.001), α-synuclein (*F(4*,10) = 13.45, *P* < 0.001), and CaM-1( *F(4*,10) = 16.16, *P* < 0.001) across experimental groups, indicating distinct treatment-driven alterations in apoptotic activity, neurodegenerative pathology, and calcium homeostasis, respectively. The expression of mRNA was doubled for caspase-3 (*P* < 0.001) and α-synuclein (*P* < 0.01) and tripled for CaM-1 (*P* < 0.001) in the MSG-treated group compared with the control group. Compared with those in the MSG group, the expression of the three genes in the groups treated with Vit D and/or N-3 PUFAs significantly decreased, restoring normal hippocampal levels akin to those in the control group. Specifically, Vit D and/or N-3 PUFAs treatment significantly decreased caspase-3 expression (*P* < 0.001) and α-synuclein expression (*P* < 0.001) for both groups when compared to the MSG group. For CaM-1, the reduction was significant with both Vit D and combination therapy (*P* < 0.001), as well as with N-3 PUFAs alone (*P* < 0.05). Notably, N-3 PUFA-treated rats exhibited a significantly higher level of CaM-1than Vit D-treated rats (*P* < 0.05). However, there was no significant difference in the effects of the individual treatments (N-3 PUFAs vs. Vit D) on either caspase-3 or α-synuclein (*P* > 0.05). Similarly, compared with that of combination therapy, there was no significant difference in the impact of individual Vit D or N-3 PUFA treatments on the hippocampal mRNA expression of these three genes (*P* > 0.05) for all.

On the other hand, one-way ANOVA revealed statistically significant differences in VDR expression across experimental groups (*F* (4, 11) = 35.91, *P* < 0.001). Although MSG administration did not significantly alter hippocampal VDR expression levels compared with those in the control group (*P* > 0.05), the Vit D treatment and combination therapy groups presented substantial increases in VDR expression compared with the MSG group and the control group *(fold change = 4.3 and 4.6* for treatments respectively, *P* < 0.001 for both). Interestingly, treatment with N-3 PUFAs alone also increased VDR expression prominently, showing a fold change of 2.9 (*P* < 0.01) compared with MSG and the control groups. Furthermore, rats treated with Vit D or combination therapy presented higher VDR levels than those treated with N-3 PUFAs alone (*P* < 0.05, *P* < 0.01) (Fig. 9A).

## Discussion

The present work aimed to highlight the potential neuroprotective effect of Vit D and N-3 PUFA supplementation against MSG-induced excitatory neurotoxicity in rats. Our findings indicate that early exposure to MSG can lead to considerable deterioration in behavioral performance and cognitive capacity at later stages, along with hippocampal inflammation and neurodegeneration, with evident overexpression of proteins related to neurodegenerative disorders. Vit D and N-3 PUFAs treatment substantially improved these deteriorations and demonstrated synergetic neuroprotective effects against hippocampal inflammation, tau accumulation, and neural apoptosis.

The behavioral tests were conducted to assess the cognitive, motor, and emotional impacts of MSG, and the effectiveness of Vit D and N-3 PUFA treatment. In the open field test (Fig. 1), MSG-exposed rats presented fewer exploratory and more anxiety-like behaviors, as indicated by decreased mobility, speed, and frequency of line crossings, along with lower frequencies of rearing behaviors, in addition to fewer visits to the central zone and shorter times recorded there. The observed reduced grooming frequencies might be considered as a state of behavioral despair, indicating decreased motivational and self-care behaviors and reflecting a depression-like state or severe stress exposure^66^. In contrast, Vit D and N-3 PUFA treatment substantially improved these measures and had an anxiolytic effect, as evidenced by increased central zone parameters, exploratory drive, and motor ability. In support of our open field results, additional N-3 PUFAs and Vit D anxiolytic, and MSG-induced anxiogenic effects were reported in the dark–light box test (Fig. 5), which revealed increased anxiety-like behaviors in MSG-exposed rats, evidenced by a longer latency to enter the light chamber and a shorter time spent in the light, while N-3 PUFA- and Vit D-treated rats presented reduced anxiety, as revealed by shorter latency to cross the light side and a longer period of light exposure. Previous animal studies have demonstrated the hypoactivity and anxiety-related behaviors associated with MSG exposure in mice^67,68^ and rats^69^, as well as the possible therapeutic anxiolytic impacts of Vit D and N-3 PUFA supplements, which are mediated via the modulation of brain neurotransmitter systems, a reduction in inflammatory cytokines, and a decrease in oxidative stress processes^70–72^. Similarly, in the NOR test (Fig. 2), the performance of MSG-exposed rats showed a tendency toward reduced exploration and a lack of preference for the novel object, indicating deficiencies in goal-oriented behavior, in line with Sachie e. al.‘s previous findings in mice treated with MSG in early life^73^. Treatments with Vit D and N-3 PUFAs improved exploration time and novelty preference, highlighting their role in enhancing cognitive flexibility. Consistent with our findings, Cutuli et al. reported that N-3 PUFA treatment reversed the novelty recognition memory impairment induced by cholinergic depletion in aged mice^74^. Similarly, Doncheva et al. reported that supplementation with Vit D3 enhances memory in rats with LPS-induced neuroinflammation, as demonstrated by the improved recognition index score in the NOR test^75^. Moreover, the MWM (Fig. 4) and T-maze tests (Fig. 3) revealed impairments in learning and memory functions in MSG-exposed rats, with prolonged latencies to locate the hidden platform or to choose the cross-arm, respectively, along with less time recorded in the target zone during the prop trial of the MWM and a low percentage of correct alternations in the T-maze, indicating impaired short-term working memory. However, treatment with Vit D and/or N-3 PUFAs substantially enhanced learning and memory performance, which was revealed by the shorter latencies recorded in both mazes, together with the longer time spent in the target zone in the MWM, as well as the achievement of a high score of correct alternation in the T-maze. These results are in line with those of González, L et al., who reported enhanced spatial and recognition memory in elderly female mice treated with N-3 PUFA^76^, and with those of Rezagholizadeh et al., who reported improved spatial and cognitive impairment induced by sleep deprivation in male rats via the injection of Vit D into the dorsal CA1 hippocampus^77^.

The present histopathological examination (Figs. 7 and 8) revealed severe neuronal degeneration in MSG-exposed rats and elevated tau protein deposition, demonstrating the neurotoxic effects of MSG exposure in early life, resulting in widespread neural cell death and tauopathy, which are key components of many neurodegenerative diseases, including AD^78,79^. Tau accumulation and its association with neurodegeneration and cognitive impairments demonstrated in the current study were reported in several previous animal^60,68,72^ and in clinical studies, where total tau (t-tau) and p-tau have been recognized as essential biomarkers for AD and have undergone successful validation through controlled, large-scale, multicenter studies^80^. In addition, t-tau protein levels in the CSF samples from humans have been linked to the degree of neuronal damage and abnormal neuronal plasticity markers in neurodegenerative diseases^81,82^.

Consistent with our findings of elevated apoptotic cell counts and significantly elevated levels of caspase-3, along with further increased CaM-1 levels (Fig. 9B, D), which reflected elevated hippocampal intracellular calcium levels in MSG-exposed rats, previous studies have linked the high intracellular calcium levels induced by excessive glutaminergic stimulation with increased caspase activation and neuronal cell death in the pathological cascades of numerous neurodegenerative disorders, including amyotrophic lateral sclerosis (ALS), PD, and AD^8^. Opland et al. also described the caspase-mediated hippocampal accumulation of aberrant tau, with subsequent synaptic dysfunction and initiation of the pathogenic cascade underlying the pathophysiology of several neurodegenerative diseases^83^. Fortunately, we found that treatment with N-3 PUFAs and/or Vit D normalized caspase-3 and attenuated CaM-1 overexpression, as well as tau accumulation. These effects support the stability of calcium signaling and homeostasis and indicate a treatment-induced neuroprotective effect against apoptosis, which is in line with previous studies showing a decrease in hippocampal apoptotic cell death and caspase activation caused by supplementation with N-3 PUFAs in aged rats, and by Vit D in rats exposed to other neurotoxic agents^84–86^. Additionally, Green et al. reported that a DHA-supplemented diet reduced Aβ accumulation and tau protein levels in transgenic mice with both Aβ plaques and tau tangles after three months of treatment^87^, although the findings from clinical research examining tau regulation by N-3 PUFA supplementation in AD patients are still controversial^88,89^. These contradictory findings could be attributed to the type of PUFA supplement, the applied dose, the duration of supplementation, and the stage of disease at the time of intervention^90^.

The elevated expression of α-synuclein may further contribute to neuronal death and cognitive deterioration^91^. MSG-treated rats presented significantly elevated α-synuclein levels in their hippocampi (Fig. 9C). This finding indicates a pathological neurotoxic response to MSG administration and suggests the formation of abnormal protein oligomers, leading to aberrant aggregation and deposition, which is a driving force in the pathogenesis of PD^92,93^, in accordance with the findings of Weston et al., which identified aggregated α-synuclein inclusions within the nucleus as predictors of impending neuronal cell death in a mouse model of PD^94^. Notably, previous cell culture studies have demonstrated that α-synuclein misfolding and aggregation might be enhanced under many circumstances, including increased intracellular calcium concentrations^95^, a condition that was established in our findings in MSG-exposed rats. In turn, the abnormal aggregation of α-synuclein has been shown previously to increase brain excitotoxicity by affecting the transport efficiency of glutamate transporters and increasing the phosphorylation of NMDARs^12^, thus generating a vicious cycle involving excessive brain glutamate, excitotoxicity, and aberrant α-synuclein aggregation. In contrast, calcipotriol, another Vit D analog, was shown to suppress calcium-dependent α-synuclein aggregation by inducing calbindin-D28k expression, which might be relevant to PD treatment^96^. These findings are in line with our results in Vit D-treated rats, which showed attenuation of MSG-induced α-synuclein overexpression in the hippocampus. Furthermore, our work revealed that supplementation with EPA and DHA PUFAs can normalize α-synuclein levels in the rat hippocampus. However, previous studies in mouse models of PD have reported conflicting results concerning the influence of DHA, particularly on α-synuclein levels in the brain. For example, a DHA-rich diet reduced abnormal α-synuclein accumulation and its associated negative outcomes^97^, while another study reported that DHA supplementation for 10 months had no significant effect on α-synuclein levels^98^. The incorporation of EPA supplementation has shown to have stronger anti-inflammatory and antioxidant effects in treating depression and neurodegenerative diseases in previous studies^99,100^ and might be a possible cause for the downregulation of MSG-induced α-synuclein overexpression observed in the N-3 PAFAs-treated rats in our study.

Our findings demonstrate that MSG exposure significantly alters the hippocampal inflammatory profile, as marked by elevated proinflammatory cytokines (IL-6, TNF-α) and decreased anti-inflammatory IL-10 (Fig. 8), that was consistent with its established neurotoxic effects in rodent models^55,56^.This cytokine dysregulation suggests a mechanistic link between MSG-induced neuroinflammation and cognitive deficits, and emphasize the critical role of neuroinflammatory cascades in mediating excitotoxicity-induced neurodegeneration^101,102^. In contrast, immunomodulatory pathways play an essential role in the neuroprotective effect of Vit D and N-3 PUFAs (EPA and DHA), as evidenced by their ability to reduce the levels of the inflammatory cytokines IL-6 and TNF-α and increase the levels of the anti-inflammatory cytokine IL-10, thereby attenuating the neuroinflammation caused by MSG in the current study^103^, which has been widely reported in previous animal studies^103–106^, and demonstrates an additional synergetic neuroprotective effect of their combined supplementation.

Primarily, a major portion of Vit D-associated neuroprotective functions is believed to be mediated through its binding to the nuclear receptor VDR, which has been widely linked to the neuroprotective, antiaging, antiapoptotic, and anti-inflammatory effects of Vit D^21,24^. Activation of the VDR by paricalcitol, a VDR-specific ligand, ameliorated pathological tau phosphorylation in the brains of transgenic mice^107^. Similarly, maxacalcitol, a Vit D analog, has been shown to lower Aβ and p-tau protein levels in rats with LPS-induced AD^108^. Notably, the present study demonstrated that both Vit D therapy alone and in combination with N-3 PUFAs significantly augmented VDR expression in hippocampal tissues by more than four-fold compared with that in control rats and the MSG group (Fig. 9A). Thus, the elevated VDR level could clarify the neuroprotective effects of Vit D, which enhances the cellular resilience against stress induced by MSG^109^. Interestingly, our findings also revealed that treatment with N-3 PUFAs alone increased VDR expression three-fold, This finding highlights the potential of N-3 PUFAs to enhance VDR expression independently, which could represent a novel way of neuroprotection, suggesting an additional mechanism for the synergistic neuroprotective effect observed in rats receiving the combined treatment, that was primarily associated with reduced neuronal apoptosis, tauopathy, and proinflammatory status in the brain. To the best of our knowledge, this is the first study to report an evident elevation in brain VDR levels upon supplementation with N-3 PUFAs in the context of induced neurotoxicity in rats. A comprehensive review of the literature did not reveal any previous studies demonstrating similar results, highlighting the novelty and significance of our findings.

The possible link between N-3 PUFAs and VDRs may include several interconnected mechanisms. N-3 PUFAs enhance VDR expression by attenuating neuroinflammation^105,110,111^, and activating the Nrf2 pathway, which mitigates oxidative stress-linked VDR suppression^112–114^. Synergistic interactions of N-3 PUFAs with multiple nuclear receptors, in particular PPAR-γ and RXR, might amplify VDR signaling further, as N-3 PUFAs promote PPAR-γ/PGC-1α pathways^115,116^and stabilize VDR-RXR heterodimers, which are critical for the VDR transcriptional activity^117,118^. Additionally, N-3 PUFAs might be able to modulate Vit D metabolism via enzymes like CYP27B1/CYP24A1, increasing bioactive Vit D availability and VDR mRNA levels^119,120^. These mechanisms can explain the complex interaction between N-3 PUFAs, Vit D, and VDRs, reflected in their effect on brain health.

Together, N-3 PUFAs (DHA/EPA) and Vit D act independently to combat MSG-induced excitotoxicity through distinct yet complementary modulation of overlapping anti-inflammatory, antioxidant, and neurotrophic pathways. Our data reveals a novel synergistic neuroprotection wherein N-3 PUFAs enhance VDR expression, thereby amplifying Vit D’s capacity to regulate gene networks involved in neuroprotection, and potentiating the anti-inflammatory and anti-apoptotic pathways, while tackling pathological protein aggregations, a common hallmark in multiple neurodegenerative diseases.

### Study limitations

The study provides a novel insight into the neuroprotective synergy of Vit D and N-3 PUFAs, nevertheless, certain aspects would require further exploration. First, the absence of control groups receiving Vit D or N-3 PUFAs alone, because the experimental design focused primarily on the MSG-induced pathologies. Integrating them into future research could help clarify their independent contributions to neuroprotection. In addition, oxidative stress-related biomarkers, such as malondialdehyde and SOD, were not assessed in the current study; their inclusion in subsequent work could deepen our understanding of how antioxidant mechanisms operate together in these interventions. Also, the four-week intervention period did not provide information about the long-term benefit sustainability. Finally, although the selected doses demonstrated major treatment efficacy, the dose-response relationships remain uncharacterized. Addressing these gaps will refine mechanistic understanding and guide translational applications.

## Conclusion

The present study concluded that treatment with Vit D and/or N-3 PUFAs substantially improved MSG-induced neurological deficits, ameliorating anxiety and cognitive impairments, with a significant reduction in hippocampal neural apoptosis and tau accumulation. Treatment further suppressed pro-inflammatory cytokines (TNF-α &IL-6) and elevated the anti-inflammatory IL-10. Both treatments clearly upregulated VDR expression, normalized caspase-3 and α-synuclein expression, and restored calcium homeostasis. The combined treatment had the most pronounced neuroprotective effect against hippocampal inflammation, tau accumulation, and neural apoptosis. These findings indicate a potential dietary approach to ameliorate MSG-induced excitotoxicity and suggest therapeutic relevance for cognitive health by counteracting neurotoxic insults. Furthermore, this synergy highlights novel avenues for developing targeted interventions to address neurodegenerative pathologies, emphasizing the translational potential of combined nutritional approaches in neuroprotection. Future studies must establish these findings in chronic models, optimize dosing regimens, and evaluate clinical translatability in different neurodegenerative disorders.

## Data Availability

The datasets used and/or analysed during the current study are available from the corresponding author on reasonable request.

## Abbreviations

Aβ: Amyloid beta
AD: Alzheimer’s disease
ALS: Amyotrophic lateral sclerosis
CaM-1: Calmodulin-1
CSF: Cerebrospinal fluid
DHA: Docosahexaenoic acid
ELISA: Enzyme-linked immunosorbent
EPA: Eicosapentaenoic acid
FDA: Food and drug administration
H_2_O2: Hydrogen peroxide
IL-6: Interleukin 6
IL-10: Interleukin 10
LTP: long-term potentiation
MSG: Monosodium glutamate
MWM: Morris water maze
NMDA: N-methyl-d-aspartate receptors
N-3 PUFAs: Omega-3 polyunsaturated fatty acids
NOR: Novel object recognition
Nrf2: Nuclear factor erythroid 2-related factor 2
NF-κB: Nuclear factor-κB
P-tau: Phosphorylated tau
PD: Parkinson’s disease
PGC-1α: Peroxisome proliferator-activated receptor-gamma coactivator-1 alpha
PPAR-γ: peroxisome proliferator-activated receptor gamma
RXR: Retinoid X receptor
SOD: Superoxide dismutase
TNF-α: Tumor necrosis factor alpha
T-tau: Total tau
VDR: Vitamin D Receptor
Vit D: Vitamin D
